# Prognostic Value of Liquid-Biopsy-Based Biomarkers in Upper Tract Urothelial Carcinoma

**DOI:** 10.3390/ijms25073695

**Published:** 2024-03-26

**Authors:** Bernat Padullés, Raquel Carrasco, Mercedes Ingelmo-Torres, Fiorella L. Roldán, Ascensión Gómez, Elena Vélez, Héctor Alfambra, Marcel Figueras, Albert Carrion, Jordi Gil-Vernet, Lourdes Mengual, Laura Izquierdo, Antonio Alcaraz

**Affiliations:** 1 Laboratori i Servei d’Urologia, Hospital Clínic de Barcelona, 08036 Barcelona, Spain; padulles@clinic.cat (B.P.); lmengual@ub.edu (L.M.);; 2 Genètica i Tumors Urològics, Institut d’Investigacions Biomèdiques August Pi i Sunyer (IDIBAPS), 08036 Barcelona, Spain; 3 Departament de Biomedicina, Facultat de Medicina I Ciències de la Salut, Universitat de Barcelona (UB), 08036 Barcelona, Spain; 4 Department of Urology, Vall d’Hebron University Hospital, 08035 Barcelona, Spain; 5 Departament de Cirurgia i Especialitats Medicoquirúrgiques, Facultat de Medicina i Ciències de la Salut, Universitat de Barcelona (UB), 08036 Barcelona, Spain

**Keywords:** biomarker, cell-free DNA, circulating tumor cells, digital PCR, liquid biopsy, prognosis, upper tract urothelial carcinoma

## Abstract

Currently, there are no reliable prognostic factors to determine which upper tract urothelial carcinoma (UTUC) patients will progress after radical nephroureterectomy (RNU). We aim to evaluate whether liquid-biopsy-based biomarkers (circulating tumor cells (CTCs), cell-free DNA (cfDNA), and circulating tumor DNA (ctDNA)) were able to predict clinical outcomes in localized UTUC patients undergoing RNU. Twenty patients were prospectively enrolled between 2021 and 2023. Two blood samples were collected before RNU and three months later. CTCs and cfDNA were isolated and evaluated using the IsoFlux system and Quant-iT PicoGreen dsDNA kit, respectively. Droplet digital PCR was performed to determine ctDNA status. Cox regression analysis was performed on CTCs, cfDNA, and ctDNA at two different follow-up time points to examine their influence on tumor progression and cancer-specific survival (CSS). During a median follow-up of 18 months, seven (35%) patients progressed and three (15%) died. Multivariate analysis demonstrated that cfDNA levels three months after RNU are a significant predictor of tumor progression (HR = 1.085; *p* = 0.006) and CSS (HR = 1.168; *p* = 0.029). No associations were found between CTC enumeration and ctDNA status with any of the clinical outcomes evaluated. The evaluation of cfDNA levels in clinical practice could improve the disease management of UTUC patients.

## 1. Introduction

Upper tract urothelial carcinoma (UTUC) is a relatively rare but challenging malignancy that affects the lining of the upper urinary tract, including the renal pelvis and ureter. It accounts for approximately 5–10% of all urothelial tumors. UTUC is considered a particularly aggressive form of urothelial cancer, often diagnosed at an advanced stage, and poses significant therapeutic difficulties due to its anatomical location and potential for early lymphatic and hematogenous spread [1]. Due to the aggressive nature of UTUC, radical nephroureterectomy (RNU) remains the “gold standard” treatment for localized tumors [2].

As researchers and clinicians strive to improve the prognosis of patients with upper urinary tract tumors, there is growing interest in identifying innovative biomarkers that can aid in early detection, monitoring treatment response, and predicting disease progression. Of note, our group has previously evaluated the role of microRNA as a prognostic biomarker in UTUC [3]. Although the results were promising, our studies included tissue samples, which implies a challenge for monitoring cancer patients owing to tissue biopsies being performed only once the mass is visible and certain tumors not being easily accessible to biopsies because of their anatomical location. These aspects may represent a challenge for tissue biopsy feasibility and may add pain and distress for the patient [4].

Several studies focusing on molecular classification have demonstrated genetically distinct groups of upper tract urothelial carcinoma (UTUC) by evaluating DNA, RNA, and protein expression. These investigations have identified five different molecular subtypes according to the mutational status of TP53, MDM2, RAS, and FGFR3, with varying gene expression, tumor location, and patient outcomes. It is currently unclear whether these subtypes will respond differently to treatment. Consequently, while these subtypes show potential, their clinical utility in daily practice is currently limited [5].

Recently, liquid biopsy has garnered considerable attention as a promising noninvasive tool with transformative potential in the field of oncology. Liquid-biopsy-based biomarkers, such as circulating tumor cells (CTCs), cell-free DNA (cfDNA), and circulating tumor DNA (ctDNA), can be easily and repeatedly obtained from cancer patients, making them optimal for real-time monitoring of tumor evolution [6].

It is believed that CTCs present in the bloodstream represent metastatic precursors that have an important role in disease invasion and progression [7]. In fact, several studies demonstrated that CTC number in peripheral blood correlates with poor outcomes [7,8,9,10]. On the other hand, high cfDNA levels in the bloodstream have been considered biomarkers of poor prognosis in different solid tumors [11,12], including urologic tumors [13,14,15,16]. Additionally, ctDNA represents a fraction of cfDNA shed by tumor cells into the bloodstream, carrying genetic information specific to the tumor’s somatic genome. The value of CTCs, cfDNA, and ctDNA has been increasingly recognized in various cancers, with studies demonstrating its utility in predicting treatment response, monitoring minimal residual disease, and detecting disease recurrence [6,17]. However, in the context of upper urinary tract tumors, the application of CTCs, cfDNA, and ctDNA as biomarkers remains relatively unexplored [7,8,9,10].

Therefore, the identification of noninvasive liquid-biopsy-based biomarkers predicting a high risk of disease recurrence and progression in UTUC patients could enhance the personalized management of UTUC patients. Here, we embarked on a comprehensive study aiming to evaluate CTC enumeration, cfDNA levels, as well as the presence of ctDNA in blood samples from UTUC patients who underwent RNU at two different follow-up time points. The integration of information from these liquid-biopsy-based biomarkers could provide a better understanding of tumor behavior and facilitate more informed therapeutic decisions. Our findings may have a significant impact on clinical care and contribute to the search for improved strategies to address this complex disease.

## 2. Results

### 2.1. Clinicopathological Features of the Cohort

In total, 20 patients (12 males, 8 females) with a median age of 71 years (range: 48–90) were analyzed. According to tumor location, nine patients presented UTUC in the renal pelvis; seven exhibited ureteral disease; while the remaining four patients presented multifocal UTUC affecting both locations.

During a median follow-up of 18 months, seven (35%) patients progressed. The mean time to progression was eight months (range 4–15 months). Among these seven patients, three presented with pT1 tumors, while the remaining four exhibited pT3 pathology. Four patients had positive lymph nodes (LN+). Two progressive patients presented only local recurrence; one patient had distant disease; while the remaining four progressive patients presented both local and distant recurrence.

During follow-up, three (15%) patients died, all due to UTUC. They all had pT3 tumors with LN+ (N1-N2). The mean cancer-specific survival (CSS) was 12 months (range 4–24 months).

A single patient, constituting 5% of the total, received neoadjuvant chemotherapy (NAC) as part of their treatment regimen due to the presence of locally advanced disease (T3N2) at the time of diagnosis. She presented distant progression eight months later.

Two patients (10%) received adjuvant chemotherapy after unfavorable pathology. Both progressed and finally died.

The clinicopathological features of the enrolled patients are summarized in Table 1.

### 2.2. CTC Enumeration for Monitoring UTUC Patients

The mean CTC number (range) at the time of RNU and three months later was 158 (47–355) and 284 (48–762) CTCs per 7.5 mL blood, respectively, for progressive patients and 136 (30–333) and 151 (33–401) CTCs per 7.5 mL blood, respectively, for nonprogressive patients.

Using the mean CTC number as the cut-off, 30% (12/40) of all blood samples had a high CTC number. Specifically, 25% and 35% of pre- and post-treatment samples, respectively, had a high CTC number.

No statistical differences were observed in CTC number at the time of RNU between progressive and nonprogressive patients. The CTC number tended to be higher in progressive than in nonprogressive patients three months after RNU, without any significative differences (*p* = 0.16).

### 2.3. cfDNA Levels during Follow-Up

The mean cfDNA levels (range) at the time of RNU and three months later were 13.92 ng/mL plasma (5.95–32.27) and 20.56 ng/mL plasma (4.9–42.88), respectively, for progressive patients and 9.29 ng/mL plasma (3.33–35) and 7.27 ng/mL plasma (1.23–18.12), respectively, for nonprogressive patients. The cfDNA quantification according to progression and CSS is summarized in the Supplementary Materials (Table S1).

Using mean cfDNA levels as the cut-off, 33% (13/40) of all plasma samples had high cfDNA values. Specifically, 30% and 35% of pre- and post-treatment samples, respectively, had a high cfDNA level.

**Figure 1:**
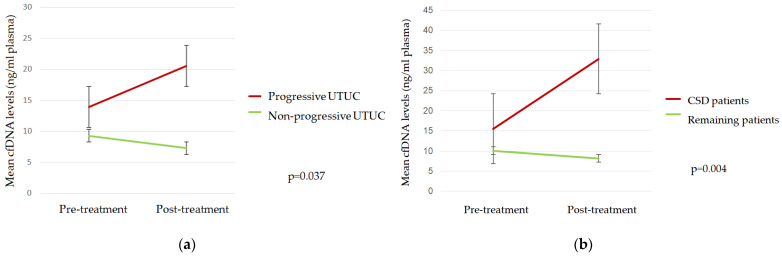
Mean cfDNA levels according to (**a**) tumor progression and (**b**) cancer-specific death (CSD).

Mean cfDNA levels three months after RNU were significantly higher in progressive than in nonprogressive patients and in those who died due to the UTUC (Figure 1).

### 2.4. ctDNA Analysis

The mean variant allele frequency (VAF) of analyzed mutations according to progression and cancer-specific death are summarized in Table 2 and Table 3.

No significant differences were found in the mean VAF of analyzed mutations (TERT c.-124C>T, TERT c.-146C>T, ATM c.1236-2A>T, and TP53 c.853G>A) in UTUC patients according to tumor progression or death occurrence.

### 2.5. Survival Analysis

CTC enumeration, cfDNA levels, ctDNA status, and clinical data at each follow-up time point were evaluated by Cox regression analysis. Univariate analysis demonstrated that the presence of positive lymph nodes (LN+; *p* = 0.008), CTC number (*p* = 0.021), and cfDNA levels (*p* = 0.006) three months after RNU were significant predictors of tumor progression. Multivariate Cox regression analysis identified cfDNA levels three months after RNU as an independent predictor of tumor progression (HR = 1.085; *p* = 0.006).

In addition, the univariate analysis identified the VAF of the TERT c.-124C>T mutation at the time of the RNU (*p* = 0.038) and cfDNA levels three months after RNU (*p* = 0.029) as significant prognostic biomarkers of CSS. Multivariate Cox regression analysis demonstrated that cfDNA levels three months after RNU are also an independent prognostic biomarker of CSS (HR = 1.168; *p* = 0.029).

**Figure 2:**
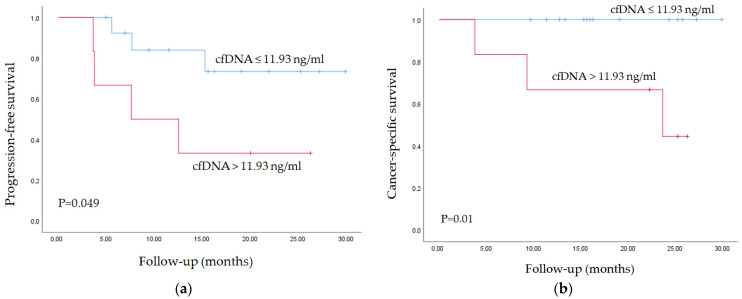
Kaplan–Meier curve for (**a**) tumor progression and (**b**) CSS according to cfDNA levels.

The mean cfDNA level (11.93 ng/mL plasma) three months after RNU was used as a cut-off point to classify patients into high- and low-risk groups for tumor progression and CSS. Figure 2 depicts the Kaplan–Meier curves generated using the selected cut-off point, proving that cfDNA levels are able to discern between two groups of UTUC patients with significantly different probabilities of tumor progression and CSS after RNU.

## 3. Discussion

Over the past few years, liquid biopsy has garnered attention in the field of oncology for its potential to monitor and control the changing course of tumor development in real time. In contrast to tumor-tissue-based methods, liquid biopsy offers a less invasive and more convenient means of obtaining samples throughout the progression of the disease [18].

In urothelial carcinoma, most attention has been focused on bladder cancer (BC) with several advances in the molecular characterization of this tumor. Some prognostic biomarkers have been identified with promising results, such as CTC number, cfDNA levels, and ctDNA status [14,15,16,19]; however, none of these biomarkers have yet been established in clinical practice.

Scant information is available about the role of liquid biopsy in UTUC. Zhang et al. performed a meta-analysis about the prognostic and diagnostic value of CTCs in urothelial carcinoma, including 30 published studies with 2161 patients; however, only six studies accepted UTUC patients and no prognostic outcomes were described [20]. Also, a 50-patient study by Nakano et al. showed the potential of ctDNA as a real-time marker of systemic therapy response in patients with metastatic UTUC [21].

To the best of our knowledge, this is the first study to enumerate blood CTCs, determine plasma cfDNA levels, and evaluate ctDNA mutations in blood samples from UTUC patients during their follow-up after RNU in order to identify prognostic biomarkers to improve UTUC management.

In the present study, we found that CTC number three months after RNU tended to distinguish two groups of patients with different probabilities of tumor progression. Although our data did not reach statistical significance, most probably for the limited series size, similar results were obtained in several studies focused on muscle-invasive BC, including our previous work [19,20,22]. Overall, these studies show that CTC number is a predictor of tumor progression and CSS in muscle-invasive bladder cancer (MIBC) during patients’ follow-up.

Here, we also found that cfDNA levels increased during patient follow-up in progressive patients and in patients who died from UTUC. Furthermore, we have demonstrated that patients with high cfDNA levels three months after RNU were at high risk of tumor progression and death. Similar findings have been described in other solid tumors. For instance, in metastatic pancreatic cancer, high cfDNA levels were strongly associated with poor outcomes [23]. There is a lack of comparable studies in UTUC; nevertheless, similar results have been found in MIBC patients. In our recent study, we identified that cfDNA levels four months after radical cystectomy was an independent biomarker of tumor progression [15].

The evaluation of cfDNA levels has several advantages over ctDNA detection: it allows for analysis in all patients regardless of their tumor’s genetic characteristics, it is less labor-intensive, has a reduced cost, and the technology used for this analysis is easily applicable in a clinical setting, given its simplicity and widespread availability in diagnostic laboratories. Nevertheless, caution is required as elevated cfDNA levels can result from non-tumor-related factors. Complementing this evaluation with the detection of specific tumor mutations in ctDNA may enhance accuracy.

In this work, we also examined the four somatic mutations previously identified in BC patients (TERT c.-124C>T, TERT c.-146C>T, ATM c.1236-2A>T, and TP53 c.853G>A) in UTUC patients. We found that the presence of mutation TERT c.-124C>T at the time of RNU is a significant prognostic biomarker of CSS. Strikingly, the presence of this mutation in BC patients was also considered a biomarker of tumor aggressivity [19].

Mutations in the promoter of the catalytic subunit of the telomerase reverse transcriptase (TERT) gene are frequently observed in several cancers and drive telomere maintenance [24,25]. In BC, TERT promoter mutations (TERT c.-124C>T and TERT c.-146C>T) are more common than any other genetic alterations and can lead to increased TERT expression and telomerase activity [26,27]. Importantly, TERT promoter mutations are associated with worse clinical outcomes in most BC studies, further highlighting the role of telomerase activation in tumor progression and recurrence [27,28,29]. Overall, these findings suggest that the presence of the TERT c.-124C > T mutation could play a crucial role in the aggressiveness of urothelial cancers.

We must acknowledge some study limitations. First, the series size is limited since UTUC is a relatively infrequent tumor. However, it should be considered that our series included a total of 40 samples from two different centers and two years of prospective recruitment. Second, we focused on two different follow-up time points only; in all probability more conclusions could be drawn if several follow-up time points had been considered. Third, the four somatic mutations analyzed here were taken from previous works of our group in BC, so this study should be considered an agnostic testing approach to UTUC. It is most likely that the knowledge of specific mutations from patient’s tissue biopsies (tumor-informed approach) would improve these results. Also, a final validation of our results in a larger and independent series would be necessary to define the real role of CTC enumeration and cfDNA monitoring in UTUC patients after RNU.

## 4. Materials and Methods

### 4.1. Patients and Samples

A total of 20 consecutive UTUC patients from two different centers (19 patients from the Hospital Clínic of Barcelona and 1 from the Vall d’Hebron Hospital) were prospectively included between 2021 and 2023. An exclusion criterion was the presence of another active neoplasm. Of these patients, 95% (19/20) had undergone RNU, and the remaining patient did not undergo any surgery due to the presence of metastatic disease at the time of diagnosis. Follow-up data were available for all patients. Tumor dissemination was controlled postoperatively via a computed tomography scan at three-monthly intervals for the first year, six-monthly intervals for the next two years, and annually thereafter. Tumors were considered progressing when relapse or distant metastasis developed during follow-up. Progression was measured from the date of RNU to progression or the final follow-up date in cases without progression.

Two 10 mL EDTA tubes of peripherical blood were collected before treatment (RNU or NAC) and three months afterward. One tube was stored at room temperature and processed to isolate CTCs within the following 24 h, and another blood tube was stored at 4 °C and processed to isolate cfDNA within the following six hours.

All methods were carried out following relevant guidelines and regulations. All patients provided written informed consent (HCB/2013/8753) before being enrolled in the study, and the study was approved by the Clinical Research Ethics Committee of the Hospital Clinic of Barcelona (HCB/2018/0026).

### 4.2. CTC Isolation and Enumeration

CTCs from blood samples were isolated via the IsoFlux system (Fluxion, Biosciences, Oakland, CA, USA) and stored at 4 °C until enumeration within the following two weeks. CTCs were fixed and immunofluorescence-stained using the CTC Enumeration Kit (Fluxion, Biosciences), following the manufacturer’s instructions. CTC enumeration was performed manually using fluorescence microscopy, according to our previous study [19].

**Figure 3:**
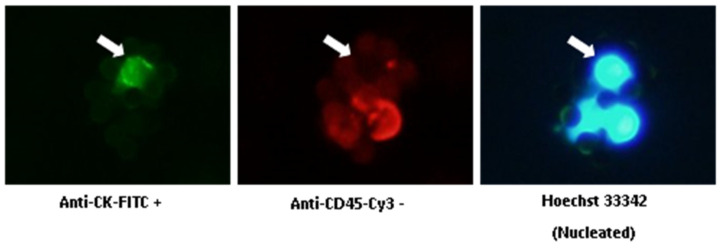
Immunofluorescent staining of CTCs. White arrows show a CTC: anti-CK-FITC positive, anti-CD45-Cy3 negative, and Hoechst 33342 positive. (Original picture.)

CTC Enumeration Kit (Fluxion, Biosciences, Oakland, CA, USA) includes anti-CK-fluorescein isothiocyanate (FITC; specific for intracellular cytokeratin of epithelial cells), anti-CD45- Indocarbocyanine (Cy3; specific for leukocytes), and Hoechst 33342 (which stains the cell nucleus). CTCs are defined as morphologically intact, CK-positive, CD45-negative, and nucleated cells (Figure 3). The number of CTCs refers to 7.5 mL blood.

### 4.3. cfDNA Isolation

Blood samples were centrifuged at 3500 rpm for 15 min at 4 °C to separate plasma, followed by plasma centrifugation at 16,000× *g* for 10 min at 4 °C to remove any remaining cells. Plasma samples were stored at −80 °C until cfDNA extraction. 

cfDNA was extracted from 2–5 mL of plasma (depending on availability) using the QIAamp Circulating Nucleic Acid kit (Qiagen, Hilden, Germany), according to the manufacturer’s instructions. cfDNA was quantified using the Quant-iT PicoGreen dsDNA assay kit (Thermo Fisher Scientific, Waltham, MA, USA) using Qubit fluorometer, according to the manufacturer’s instructions. 

### 4.4. Droplet Digital PCR

Droplet digital PCR (ddPCR) was performed using the QX200 Droplet Digital PCR system (Bio-Rad, Watford, UK) following the manufacturer’s instructions. The four somatic mutations previously identified in bladder urothelial carcinoma (TERT c.-124C>T, TERT c.-146C>T, ATM c.1236-2A>T, and TP53 c.853G>A) [14] were selected to be analyzed in a total of 40 plasma cfDNA samples from the 20 UTUC patients enrolled in this study. The four mutations were analyzed in all patients (tumor-agnostic testing). Briefly, the reactions for custom assays (*ATM* and *TERT* c.-124C>T) were performed with 11 µL SuperMix no dUTP (Bio-Rad), 0.5 µL forward + reverse primers (40 µM), 1.1 µL HEX probe (5 µM), 1.1 uL FAM probe (5 µM), and 8.3 µL cfDNA sample. The reactions for commercial assays (TP53 and TERT c.-146C>T) were performed with 11 µL SuperMix no dUTP (Bio-Rad), 1.1 µL assay (20 µM), 1.6 µL H2O, and 8.3 µL cfDNA sample. PCR was performed as follows: 95 °C for 10 min then 40 cycles (except for TERT c.-124C>T, which was 50 cycles) of 94 °C for 30 s, the specific assay annealing temperature for 1 min (60 °C for *TERT* c.-124C>T, 59° C for *TERT* c.-146C>T, 56.5 °C for *ATM*, and 57.5 °C for *TP53*), followed by 98 °C for 10 min, and a final hold at 4 °C. Each reaction was performed with a negative control (10,000 copies wt allele + 0 copy mutant allele) and positive control (10,000 copies wt allele + 10 copies mutant allele). The limit of detection (LOD) of the mutant allele was 0.08% for TERT c.-124C>T and TERT c.-146C>T, 0.05% for ATM c.1236-2A>T, and 0.1% for TP53 c.853G>A [15].

The VAF was calculated as the number of droplets positive with mutant amplicon, divided by total droplets positive with amplicon (wild-type and mutant). ctDNA was defined as detectable if the VAF was above the LOD of each mutant amplicon. Data analysis was carried out using QuantaSoft Analysis Pro Software, version 1.0 (Bio-Rad Laboratories, Hercules, CA, USA).

### 4.5. Statistical Analysis

Differences in CTC enumeration, cfDNA levels, and ctDNA status between pre- and post-treatment were analyzed using the Mann–Whitney U-test for independent samples and the Friedman test for repeated measures. A ctDNA sample was considered positive (or detectable) if at least one of the four mutations had a VAF higher than 35% of all VAF values. This 35% VAF value corresponded to 0.16 for TERT c.-124C>T, 0.15 for TERT c.-146C>T, 0.07 for ATM c.1236-2A>T, and 0.54 for TP53 c.853G>A. CTC number and cfDNA levels were dichotomized using their mean value: 171 CTCs and 11.42 ng/mL plasma, respectively. These mean values were used as cut-offs to discriminate between high and low CTC numbers and cfDNA levels, respectively. Cox regression analysis was performed on CTC enumeration, cfDNA levels, and ctDNA status at two different follow-up time points to examine their influence on tumor progression and CSS. Statistical significance was established at a *p*-value of 0.05. Kaplan–Meier curves were generated and compared using log-rank tests. All analyses were carried out with the SPSS software package (IMB SPSS Statistics 25).

## 5. Conclusions

Our results suggest that cfDNA levels three months after RNU are an independent prognostic biomarker of tumor progression and CSS in UTUC patients who underwent RNU. The implementation of cfDNA analysis in clinical management could have an impact on disease prognosis, since patients could benefit from early treatment.

## Figures and Tables

**Table 1:** Clinicopathological features of UTUC patients enrolled in the study.

	Total UTUC (N = 20)	Progressive UTUC (N = 7)	Non-Progressive UTUC (N = 13)
Gender, n (%)			
Male	12 (60)	4 (57)	8 (62)
Female	8 (40)	3 (43)	5 (38)
Tumor location, n (%)			
Pelvis	9 (45)	3 (43)	6 (46)
Ureter	7 (35)	1 (14)	6 (46)
Both	4 (20)	3 (43)	1 (8)
Pathological Stage, n (%)			
pTa	3 (15)	-	3 (23)
pT1	7 (35)	3 (43)	4 (31)
pT2	4 (20)	-	4 (31)
pT3	6 (30)	4 (57)	2 (15)
pT4	-	-	-
Histological Grade, n (%)			
Low	6 (30)	0 (0)	6 (46)
High	14 (70)	7 (100)	7 (54)
Metastasis, n (%)			
Local	2 (10)	2 (29)	-
Distant	1 (5)	1 (14)	-
Local + distant	4 (20)	4 (57)	-
Nodes, n (%)	4 (57)	4 (57)	-
Chemotherapy, n (%)			
Adjuvant	2 (10)	2 (29)	-
Neoadjuvant	1 (5)	1 (14)	-

**Table 2:** Mean VAF according to tumor progression.

	Pre-Treatment VAF	Post-Treatment VAF	*p*-Value
**Progressive UTUC**			
*TERT* c.-124C>T	81%	18%	0.19
*TERT* c.-146C>T	8%	3%	0.24
*ATM* c.1236-2A>T	1%	0%	0.31
*TP53* c.853G>A	6%	40%	0.11
**Nonprogressive UTUC**			
*TERT* c.-124C>T	1%	7%	0.21
*TERT* c.-146C>T	19%	10%	0.79
*ATM* c.1236-2A>T	6%	7%	0.60
*TP53* c.853G>A	82%	86%	0.12

**Table 3:** Mean VAF according to cancer-specific survival (CSS).

	Pre-Treatment VAF	Post-Treatment VAF	*p*-Value
**Cancer-specific death**			
*TERT* c.-124C>T	159%	34%	0.09
*TERT* c.-146C>T	8%	0%	0.23
*ATM* c.1236-2A>T	1%	1%	0.55
*TP53* c.853G>A	83%	8%	0.11
**Remaining patients**			
*TERT* c.-124C>T	12%	7%	0.15
*TERT* c.-146C>T	16%	9%	0.33
*ATM* c.1236-2A>T	5%	5%	0.41
*TP53* c.853G>A	73%	81%	0.20

## Data Availability

Data are available upon reasonable request.

## Supplementary Materials

The following supporting information can be downloaded at: https://www.mdpi.com/article/10.3390/ijms25073695/s1.
