# Supplementary material for: Prognostic Value of Liquid-Biopsy-Based Biomarkers in Upper Tract Urothelial Carcinoma
Source: Int J Mol Sci. 2024 Mar 26;25(7):3695. doi: 10.3390/ijms25073695 (PMC11012136; doi:10.3390/ijms25073695)
Supplement: Supplementary file 1 [file ijms-25-03695-s001.zip › ijms-2914576-supplementary.pdf]

International Journal of
Molecular Sciences

MDPI

TABLES – SUPPLEMENTARY MATERIAL

Table S1. cfDNA levels quantification.

Patient number	Pre-surgery cfDNA levels (ng/ml)	Post-surgery cfDNA levels (ng/ml)	Progressive (P)	Deceased (D)
1	11,69	6,75		
2	35	18,12		
3	8,12	18,9	P	D
4	32,27	37,03	P	D
5	4,83	3,19		
6	3,94	8,33		
7	9,28	8,89	P	
8	17,85	26,04	P	
9	5,81	4,06		
10	6,65	5,53		
11	10,06	15,67		
12	4,06	6,40		
13	4,40	1,23		
14	8,91	2,33		
15	3,33	3,94		
16	5,95	5,25	P	
17	9,38	7,2		
18	6,24	42,88	P	D
19	12,72	11,81		
20	17,76	4,9	P	
